# A prediction model for left ventricular thrombus persistence/recurrence: based on a prospective study and a retrospective study

**DOI:** 10.1186/s12959-023-00488-1

**Published:** 2023-05-01

**Authors:** Qing Yang, Xin Quan, Chuangshi Wang, Litian Yu, Yanmin Yang, Jun Zhu, Yan Liang

**Affiliations:** 1 National Clinical Research Center of Cardiovascular Diseases, Fuwai Hospital, National Center for Cardiovascular Diseases, Chinese Academy of Medical Sciences and Peking Union Medical College, Beijing, 100037 China; 2 Department of Echocardiographic, Fuwai Hospital, National Center for Cardiovascular Diseases, Chinese Academy of Medical Sciences and Peking Union Medical College, Beijing, 100037 China; 3 Medical Research and Biometrics Center, Fuwai Hospital, National Center for Cardiovascular Diseases, Peking Union Medical College, Chinese Academy of Medical Sciences, Room 101-106, Block A, Shilong West Road, Mentougou District, Beijing, China; 4 Intensive Care Center, Fuwai Hospital, National Center for Cardiovascular Diseases, Chinese Academy of Medical Sciences and Peking Union Medical College, Beijing, 100037 China; 5 Emergency Center, Fuwai Hospital, National Center for Cardiovascular Diseases, Chinese Academy of Medical Sciences and Peking Union Medical College, Beijing, 100037 China

**Keywords:** Left ventricular thrombus, Prediction model, Thrombus persistence/recurrence

## Abstract

**Background:**

It remains unknown whether anticoagulation for persistent left ventricular (LV) thrombus should be continued indefinitely. Identifying patients with a high risk of thrombus unresolved may be helpful to determine the optimum anticoagulation duration. This study aimed to develop a prediction model to forecast thrombus persistence or recurrence in patients with LV thrombus.

**Methods:**

We enrolled patients prospectively from 2020 to 2022 and retrospectively from 2013 to 2019 at the National Center of Cardiovascular Diseases of China. The two cohorts were then combined to derive predictive models of thrombus persistence/recurrence. The primary study comprised patients who received systemic oral anticoagulants and had imaging records available at the end of a 3-month follow-up period. The Lasso regression algorithm and the logistic regression were performed to select independent predictors. The calibration curve was generated and a nomogram risk prediction model was applied as a risk stratification tool.

**Results:**

A total of 172 (64 in the prospective cohort and 108 in the retrospective cohort) patients were included, with 124 patients in a training set and 48 patients in a validation set. Six predictors were incorporated into the multivariate logistic regression prediction model. The area under the receiving operating characteristic was 0.852 in the training set and 0.631 in the validation set. Patients with protuberant thrombus and higher baseline D-dimer levels had a reduced risk of persistence/recurrence (OR 0.17, 95% CI 0.03–0.69, *P* = 0.025; OR 0.67, 95% CI 0.43–0.91, *P* = 0.030, separately), whereas thicker thrombus was linked to an increased rate of persistent thrombus (OR 1.11, 95% CI 1.05–1.20, *P* = 0.002). Additionally, patients with diverse diagnoses or receiving different antiplatelet treatments had different rates of LV thrombus persistence/recurrence at 3 months.

**Conclusions:**

This prediction model provides tools to forecast the occurrence of persistent/recurrent thrombus and allows the identification of characteristics associated with unresolved thrombus. To validate the model and determine the duration of anticoagulation in patients with persistent thrombus, prospective randomized trials are necessary.

**Supplementary Information:**

The online version contains supplementary material available at 10.1186/s12959-023-00488-1.

## Introduction

Left ventricular (LV) thrombus has been associated with up to 22% risk of embolization in the past [1]. Guidelines recommend that patients with ischemic cardiomyopathy (ICM) and LV thrombus should receive oral anticoagulation for 3 months [2], while patients with nonischemic cardiomyopathy (NICM) should be treated with oral anticoagulation for at least 3–6 months, to reduce the risk of stroke or systemic embolism events [3]. Based on the 2022 statement for LV thrombus, anticoagulation should be discontinued if patients had a resolution of LV thrombus with left ventricular ejection fraction (LVEF) improving to > 35% or major bleeding occurring [3]. However, considering patients with persistent thrombus despite anticoagulation, there are no sufficient study data to determine whether anticoagulation should be continued indefinitely. Therefore, identifying patients with a high risk of thrombus unresolved may be helpful to provide evidence on the management of anticoagulation.

Depending on a prospective trial and a retrospective study, we aimed to investigate potential factors associated with thrombus unresolved in the population of patients who received oral anticoagulation for 3 months and then provide a prediction model to determine the risk of thrombus persistence or recurrence in patients with LV thrombus.

## Methods

### Study design and patient population

This study was derived from two studies, including a prospective study named R-DISSOLVE (ClinicalTrials.gov: NCT04970381) and a retrospective registry study (ClinicalTrials.gov: NCT 05,006,677), carried out at Fuwai Hospital, National Center of Cardiovascular Diseases in China. R-DISSOLVE was an interventional, single-arm, open-label, investigator-initiated study between October 2020 and April 2022. The retrospective study collected data from June 2013 to December 2019 by using electronic medical records. The two study protocols were developed separately and approved by the ethics committee at the participating center. The combined study was reported in accordance with the TRIPOD checklist [4].

In the prospective study, patients with LV thrombus for less than 3 months and with systemic anticoagulation of less than 1 month were enrolled. Patients with inherited or acquired thrombophilia (e.g., antiphospholipid syndrome) were excluded since the risk of thrombus persistence/recurrence in these patients was established on a unique pathophysiological mechanism. The inclusion and exclusion criteria in the retrospective study were similar to those used for the prospective one. To increase the sample size [5–7], we pooled the data from the two studies to create a combined study, which was then used to develop a statistical model of thrombus persistence/recurrence prediction. Only patients having imaging records at follow-up visits and having continued systemic oral anticoagulation for at least 3 months were eligible for the primary analysis, as evidenced by objective data such as prescriptions from cardiologists.

### Definitions

LV thrombus was defined as an abnormal echo mass in the left ventricular cavity, whose edge was different from the left ventricular endocardium [8]. In the prospective trial, thrombus was quantified using contrast-enhanced echocardiography (CE) at baseline and follow-up visits, whereas in the retrospective study, thrombus confirmed by transthoracic echocardiography (TTE), computer tomography (CT), or cardiac magnetic resonance imaging (CMR) was obtained. When a thrombus was detected, its morphology was categorized as either mural (if its borders are generally continuous with the adjacent endocardium) or protuberant (if its borders are distinct from the adjacent endocardium and protrude into the ventricular cavity) [3].

The primary endpoint of this combined study was the LV thrombus persistence/recurrence rate at 3 months confirmed by image techniques. The thrombus persistence was defined as the presence of a thrombus at 3 months that was comparable to the one at baseline. The thrombus recurrence was defined as the presence of thrombus at 3 months following negative images from baseline to 3 months. Safety outcomes included major bleeding according to the International Society on Thrombosis and Haemostasis [ISTH] [9] criteria and clinically related non-major hemorrhage events [10]. Additionally, stroke or embolic events were collected at 3 months.

### Model development

A total of 44 variables were collected in the initial database. The data from each study were randomly split into a training set (70% of the sample) and a validation set (30% of the sample). The final sets were created by merging the training set and validation set from each study. The following five stages summarize the process of developing and validating prediction models. *First,* create the prediction models. The significant variables from the univariate logistic analysis and variables of interest were combined to generate Model 1. Model 2 was created using the Lasso regression algorithm to identify additional potential variables associated with prognosis. *Second,* assess model discrimination ability by the mean area under the receiver operating characteristic curve (AUC). *Third,* assess model calibration to compare the predicted with the actual rates of thrombus persistence/recurrence [11]. *Fourth,* assess the clinical effectiveness of models by the decision curve analysis (DCA) to quantitatively depict the net benefit of clinical decisions. *Finally,* draw a nomogram to visualize prediction models, which can relatively calculate the risk of thrombus persistence/recurrence at 3 months in each patient by calculating the total score of each independent factor.

### Statistics analysis

Descriptive statistics were computed using the CBCgrps-Package in R [12]. Mean (standard deviation, SD) or median (interquartile range, IQR) for continuous variables and frequency (percentage) for categorical variables were reported. The Pearson chi-squared test or the Fisher exact test was performed for categorical data, and the Student unpaired *t*-test or the Mann–Whitney U test was applied to compare continuous variables. Odds ratios (OR) and 95% confidence intervals (CI) were estimated using regression models. To address missing data for predictor variables, multiple imputations by chained equations with predictive mean matching (MICE-Package in R) were used to create five sets of imputed data. The car package in R was used to detect collinearity between variables, and a variance inflation factor < 10 was tolerated. In addition, a restricted cubic spline curve was used between the continuous variables and the primary outcome. All analyses were scheduled for completion with R Studio and R, Version 3.5.1 (The R Project for Statistical Computing, Vienna, Austria).

## Results

### Study population

**Fig. 1:**
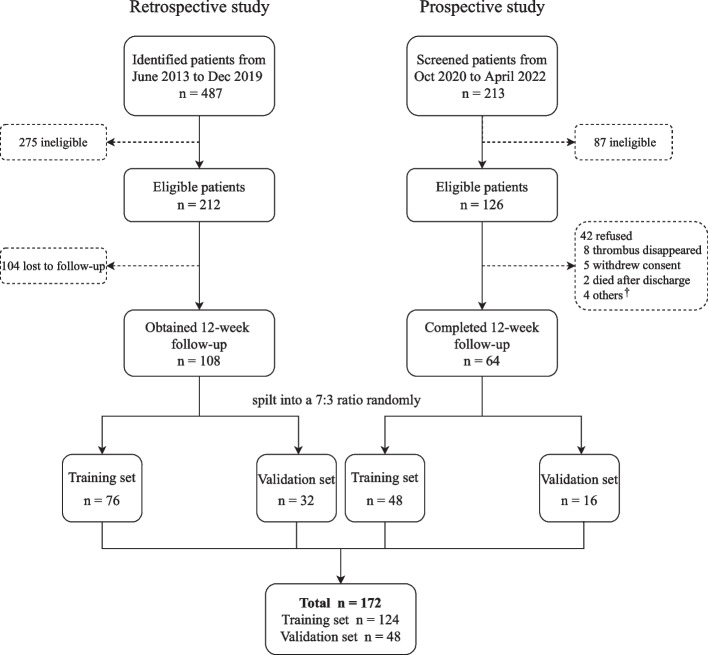
Study flow diagram. A total of 172 patients were included in our analysis, which was split into a training set (*n* = 124, 70% of the sample) and a validation set (*n* = 48, 30% of the sample). ^†^Others include 5 patients suspected of antiphospholipid antibody syndrome (*n* = 2) or thrombophilia (*n* = 3) at discharge. N, numbers of patients

A total of 487 patients and 213 patients were screened in the retrospective and prospective study respectively (Fig. 1). At a 3 months follow-up, 108 patients and 64 patients separately were included in the combined study. Patients from the two groups were divided into a training set and a validation set with a 7:3 ratio. The final training group and validation group, respectively, comprised 124 patients and 48 patients.

The average age of the entire population was 49.8 years, and 143 out of 172 (83%) patients were male. Ischemic cardiomyopathy (ICM) was the leading underlying cause (46.5%). The median LVEF level was 30% and 126 (73.3%) patients had a mural thrombus. Regarding anticoagulation therapy, 123 (71.5%) patients received non-vitamin K antagonist oral anticoagulants (NOACs) therapy, while the remaining were with warfarin. No patients switched the type of oral anticoagulation within 3 months. Of the 123 patients, 99.2% were given rivaroxaban (49.2% with a reduced dose). In addition, 105 (61%) patients received no antiplatelet, while 44 patients received mono antiplatelet agents (36 on clopidogrel and 8 on aspirin) and 23 patients received dual antiplatelet therapy (22 on aspirin plus clopidogrel and 1 on aspirin plus ticagrelor) in the combination with anticoagulation. Baseline characteristics were presented in Table 1 and no significant differences were observed between the training group and the validation group.

**Table 1:** Baseline characteristics of patients with LV thrombus

	**Total** **(*N* = 172)**	**Training group** **(*N* = 124)**	**Validation group** **(*N* = 48)**	***P-*value**
Age, y	49.8 ± 14.3	50.2 ± 14.6	48.7 ± 13.5	0.526
Male, n (%)	143 (83.1)	109 (87.9)	34 (70.8)	0.014
BMI, kg/m^2^	25.2 ± 3.9	25.0 ± 3.9	25.6 ± 3.9	0.391
Systolic blood pressure, mmHg	117 ± 19	117 ± 19	117 ± 18	0.993
Diastolic blood pressure, mmHg	78 ± 14	78 ± 13	78 ± 16	0.810
Heart rate, bpm	83 ± 18	83 ± 19	83 ± 16	0.890
**Diagnosis, n (%)**				0.715
ICM	80 (46.5)	59 (47.6)	21 (43.8)	
DCM	56 (32.6)	41 (33.1)	15 (31.2)	
Others^a^	36 (20.9)	24 (19.4)	12 (25)	
**Medical history, n (%)**				
Atrial fibrillation	11 (6.4)	10 (8.1)	1 (2.1)	0.295
Heart failure	101 (58.7)	74 (59.7)	27 (56.2)	0.813
Hypertension	79 (45.9)	57 (46)	22 (45.8)	1.000
Diabetes mellitus	35 (20.3)	28 (22.6)	7 (14.6)	0.338
Hyperlipdimia	94 (54.7)	66 (53.2)	28 (58.3)	0.665
Chronic kidney disease	12 (7)	10 (8.1)	2 (4.2)	0.514
Embolism	23 (13.4)	19 (15.3)	4 (8.3)	0.338
**Imaging measurements**				
LVEF, %	30 (23, 40)	30 (22, 40)	30 (25, 39)	0.628
LV end-diastolic diameter, mm	62 (56, 68)	62 (56, 69)	60 (54, 68)	0.346
Amount of thrombus, n (%)				0.340
1	142 (82.6)	105 (84.7)	37 (77.1)	
≥ 2	30 (17.4)	19 (15.3)	11 (22.9)	
Thrombus morphology, n (%)				1.000
Mural	126 (73.3)	91 (73.4)	35 (72.9)	
Protuberant	46 (26.7)	33 (26.6)	13 (27.1)	
Size of LV thrombi, mm				
Diameter	22 (16, 32)	22 (15, 32)	23 (19, 33)	0.428
Thickness	12.5 (9, 17)	12 (9, 17)	13 (9, 15)	0.933
Spontaneous echo contrast, n (%)	19 (11)	13 (10.5)	6 (12.5)	0.915
Regional wall motion abnormality, n (%)	74 (43)	55 (44.4)	19 (39.6)	0.693
Ventricular aneurysm, n (%)	55 (32)	38 (30.6)	17 (35.4)	0.675
**Laboratory test**				
D-dimer, ug/ml	1.1 (0.4, 2.2)	1.2 (0.4, 2.2)	1.0 (0.4, 2.3)	0.808
FDP, ug/ml	3.5 (2.5, 6.3)	3.5 (2.5, 6.13)	3.6 (2.5, 7.1)	0.555
C-reactive protein, mg/L	6.3 (3.0, 15.8)	6.3 (3.0, 14.7)	6.3 (3.1, 20.5)	0.868
APTT, S	38.3 (34.3, 43.0)	38.0 (34.5, 43.1)	38.8 (33.8, 42.0)	0.489
PT, S	14.2 (13.2, 15.5)	14.2 (13.3, 15.5)	13.9 (13.0, 15.4)	0.352
Creatinine clearance, n (%)				0.740
< 50 mL/min	151 (87.8)	110 (88.7)	41 (85.4)	
≥ 50 mL/min	21 (12.2)	14 (11.3)	7 (14.6)	
NT-proBNP, pg/ml	1945 (758, 4937)	1786 (633, 4937)	2216 (969, 4826)	0.38
**Treatment**				
Antiplatelet therapy, n (%)				0.085
None	105 (61)	72 (58.1)	33 (68.8)	
Mono	44 (25.6)	31 (25)	13 (27.1)	
Dual	23 (13.4)	21 (16.9)	2 (4.2)	
Heparin, n (%)	149 (48)	111 (50)	38 (41)	0.188
Anticoagulation therapy, n (%)				0.492
Warfarin	49 (28.5)	33 (26.6)	16 (33.3)	
NOACs	123 (71.5)	91 (73.4)	32 (66.7)	

Variables are presented as n (%), mean ± SD, and median (IQR)

*Abbreviations*: *LV* Left ventricular, *N* Numbers of patients, *SD* Standard deviation, *IQR* Interquartile range, *BMI* Body mass index, *ICM* Ischemic cardiomyopathy, *DCM* Dilated cardiomyopathy, *LVEF* Left ventricular ejection fraction, *FDP* Fibrin degradation products, *APTT* Activated partial thromboplastin time, *PT* Prothrombin time, *NT-proBNP* N-Terminal pro-brain natriuretic peptide, *NOACs* Non-vitamin K antagonist oral anticoagulants

^a^Other diagnoses include hypertensive heart disease (*n* = 13), inflammatory cardiomyopathy (*n* = 2), heart failure (*n* = 3), restrictive cardiomyopathy (*n* = 2), hypertrophic cardiomyopathy (*n* = 4), noncompaction of ventricular myocardium (*n* = 2), and valvular heart disease (*n* = 2), arrhythmogenic right ventricular cardiomyopathy (*n* = 3), myocarditis (*n* = 1, as follows), chemotherapy-induced cardiomyopathy, peripartum cardiomyopathy, infective endocarditis, metabolic cardiomyopathy, and cardiac arrhythmias

### Development of two models via univariate and Lasso regression

**Fig. 2:**
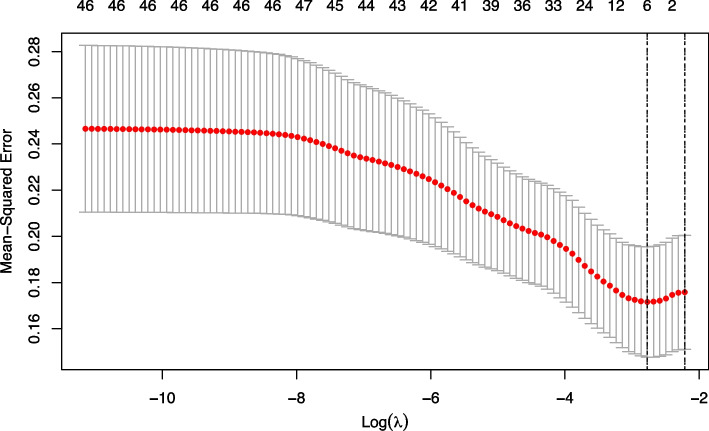
Variable selection in the LASSO regression using ten-fold cross-validation. Dotted vertical lines were drawn at the optimal values by using the minimum criteria and the 1-SE criteria. A Lambda value of 0.000011 was chosen according to ten-fold cross-validation. SE, standard error

We collected 44 characteristics in the primary database. A total of 11 variables with a *P* value < 0.10 were selected from the univariable analysis (Table 2). By including an additional four variables of interest (diagnosis, LVEF, anticoagulation therapy, and D-dimer levels) [13–16], six predictors were finally incorporated into Model 1- diagnosis, antiplatelet therapy, the thickness of thrombi, thrombus morphology, ventricular aneurysm, and D-dimer levels (Table 3). On the other hand, ten-fold cross-validation of the Lasso coefficient profiles of 44 characteristics led to the selection of Lambda = 0.000011 as the minimum criterion for the Lasso regression (Figure S1). A total of ten variables remained after variable selection using the LASSO penalty (Fig. 2), six of which were components of Model 2 in the multivariable logistic analysis (Table 3). They were listed as follows: diagnosis, antiplatelet therapy, the thickness of thrombi, thrombus morphology, spontaneous echo contrast, and D-dimer levels.

**Table 2:** Univariate analysis associated with LV thrombus persistence/recurrence at 3 months in the training group

Variable	Thrombus resolved (*N* = 97)	Thrombus unresolved (*N* = 27)	Univariable	
			**OR (95% CI)**	***P-*value**
Age	49.6 ± 14.5	52.5 ± 14.8	1.01 (0.98–1.04)	0.355
Male	84 (86.6)	25 (92.6)	1.93 (0.41–9.15)	0.405
BMI	24.6 ± 3.6	26.4 ± 4.7	1.13 (1.01–1.26)	0.039
Systolic blood pressure	115 ± 18	122 ± 21	1.02 (1.00–1.04)	0.080
Diastolic blood pressure	78 ± 14	77 ± 12	1.00 (0.96–1.03)	0.865
Heart rate	84 ± 20	77 ± 14	0.98 (0.95–1.00)	0.089
**Diagnosis, n (%)**				
ICM	43 (44.3)	16 (59.3)	Reference	
DCM	35 (36.1)	6 (22.2)	0.46 (0.16–1.30)	0.144
Others	19 (19.6)	5 (18.5)	0.71 (0.23–2.21)	0.552
**Medical history**				
Atrial fibrillation	10 (10.3)	0 (0)	NA	0.990
Heart failure	61 (62.9)	13 (48.1)	0.55 (0.23–1.30)	0.170
Hypertension	45 (46.4)	12 (44.4)	0.92 (0.39–2.18)	0.858
Diabetes mellitus	21 (21.6)	7 (25.9)	1.27 (0.47–3.40)	0.639
Hyperlipdimia	51 (52.6)	15 (55.6)	1.13 (0.48–2.66)	0.784
Chronic kidney disease	8 (8.2)	2 (7.4)	0.89 (0.18–4.46)	0.887
Embolism	15 (15.5)	4 (14.8)	0.95 (0.29–3.14)	0.934
**Imaging measurements**				
LVEF	28 (21, 40)	37 (27, 40)	1.03 (1.00–1.07)	0.061
LV end-diastolic diameter	63 (56, 69)	61 (56, 65)	1.00 (0.96–1.05)	0.865
Amount of thrombus				
1	81 (83.5)	24 (88.9)	Reference	
≥ 2	16 (16.5)	3 (11.1)	0.63 (0.17–2.36)	0.495
Thrombus morphology				
Mural	67 (69.1)	24 (88.9)	Reference	
Protuberant	30 (30.9)	3 (11.1)	0.28 (0.08–1.00)	0.050
Size of LV thrombi				
Diameter	20 (14, 31)	28 (21, 35)	1.03 (1.00–1.06)	0.083
Thickness	11 (9, 16)	16 (10, 23)	1.06 (1.01–1.12)	0.013
Spontaneous echo contrast	13 (13.4)	0 (0)	NA	0.988
Regional wall motion abnormality	38 (39.2)	17 (63)	2.64 (1.09–6.38)	0.031
Ventricular aneurysm	24 (24.7)	14 (51.9)	3.28 (1.35–7.93)	0.009
**Laboratory test**				
D-dimer	1.2 (0.4, 2.3)	0.8 (0.3, 1.6)	0.83 (0.62–1.10)	0.187
FDP	3.6 (2.5, 6.3)	3.1 (2.5, 4.3)	0.96 (0.89–1.03)	0.259
C-reactive protein	6.11 (3.05, 14)	8.12 (2.7, 18.8)	1.00 (0.99–1.02)	0.625
APTT	38.1 (34.3, 43.2)	37.9 (35.4, 42.6)	1.01 (0.95–1.08)	0.691
PT	14.2 (13.2, 15.3)	14.2 (13.4, 16.0)	0.96 (0.85–1.09)	0.547
Creatinine clearance				
< 50 mL/min	85 (87.6)	25 (92.6)	Reference	
≥ 50 mL/min	12 (12.4)	2 (7.4)	0.57 (0.12–2.70)	0.476
NT-proBNP	2063 (857, 4932)	1165 (412, 4916)	1.00 (1.00, 1.00)	0.809
**Treatment**				
Antiplatelet therapy				
None	63 (64.9)	9 (33.3)	Reference	
Mono	21 (21.6)	10 (37)	3.33 (1.19–9.31)	0.022
Dual	13 (13.4)	8 (29.6)	4.31 (1.40–13.27)	0.011
Heparin	61 (62.9)	15 (55.6)	0.74 (0.31–1.75)	0.490
Anticoagulation therapy				
Warfarin	23 (23.7)	10 (37)	Reference	
NOACs	74 (76.3)	17 (63)	0.53 (0.21–1.31)	0.170

Variables are presented as n (%), mean ± SD, and median (IQR)

*Abbreviations*: *LV* Left ventricular, *N* Numbers of patients, *SD* Standard deviation, *IQR* Interquartile range, *BMI* Body mass index, *ICM* Ischemic cardiomyopathy, *DCM* Dilated cardiomyopathy, *LVEF* Left ventricular ejection fraction, *FDP* Fibrin degradation products, *APTT* Activated partial thromboplastin time, *PT* Prothrombin time, *NT-proBNP* N-Terminal pro-brain natriuretic peptide, *NOACs* Non-vitamin K antagonist oral anticoagulants

**Table 3:** Two models for LV thrombus persistence/recurrence at 3 months^a^

Predcitors	Model 1		Model 2	
	**OR (95% CI)**	***P-*value**	**OR (95% CI)**	***P-*value**
Diagnosis				
DCM vs ICM	10.04 (1.25–130.15)	0.046	4.95 (0.79–50.01)	0.118
Others vs ICM	15.61 (1.81–204.30)	0.020	8.04 (1.10–86.61)	0.055
Antiplatelet therapy				
mono vs none	22.46 (3.63–230.79)	0.003	20.34 (3.53–191.32)	0.002
dual vs none	17.47 (2.26–215.22)	0.012	17.26 (2.38–193.29)	0.010
Thickness of thrombi	1.11 (1.05–1.20)	0.002	1.13 (1.05–1.24)	0.003
Thrombus morphology				
protuberant vs mural	0.17 (0.03–0.69)	0.025	0.24 (0.04–0.97)	0.068
Spontaneous echo contrast	-	-	NA	0.991
Ventricular aneurysm	2.54 (0.73–9.63)	0.151	-	-
D-dimer	0.67 (0.43–0.91)	0.030	0.70 (0.44–0.94)	0.058

*Abbreviations*: *LV* Left ventricular, *OR* Odds ratio, *CI* Confidence interval, *ICM* Ischemic cardiomyopathy, *DCM* Dilated cardiomyopathy

^a^Model 1 and Model 2 are conducted via multivariate analysis based on the univariate logistic regression and Lasso regression separately

**Fig. 3:**
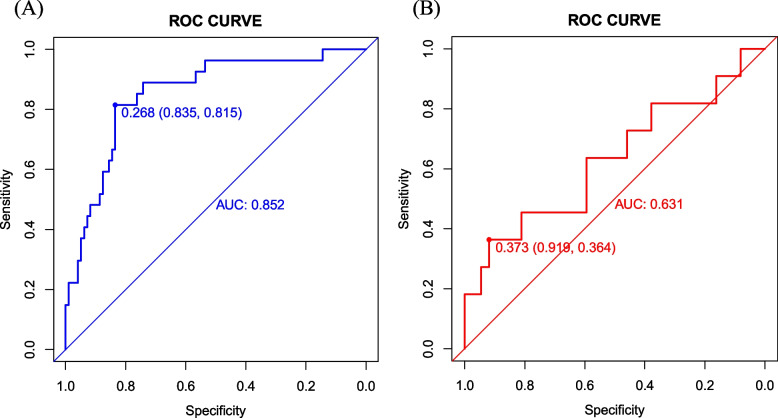
ROC curves of Model 1 for predicting the risk of LV thrombus persistence/recurrence at 3 months. **A** Training set. **B** Validation set. The blue curve represents the model discrimination ability of Model 1 in the training set (AUC 0.852, 95% CI 0.771–0.933). The red curve represents the model discrimination ability of Model 1 in the validation set (AUC 0.631, 95% CI 0.421–0.842). The point in the curve represents the optimal threshold along with the corresponding specificity and sensitivity respectively. LV, left ventricular; ROC, receiver operating characteristic; AUC, area under the ROC curve

**Fig. 4:**
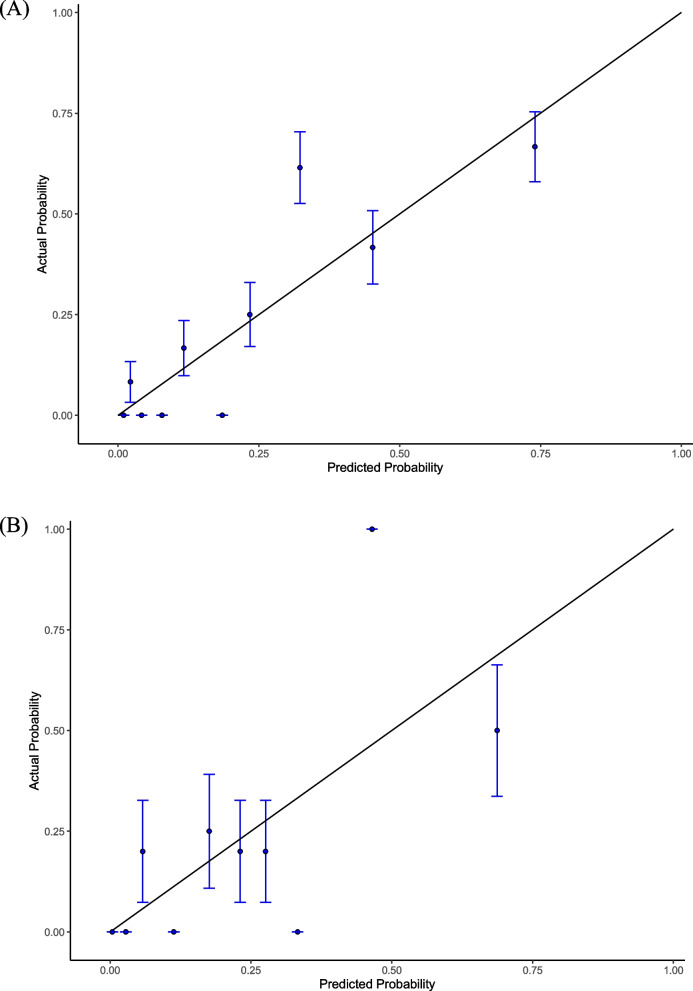
Calibration plots for predicting the risk of LV thrombus persistence/recurrence at 3 months in Model 1. **A** Training set. **B** Validation set. X-axis: predicted thrombus persistence/recurrence risk; Y-axis: actual thrombus persistence/recurrence rate. Estimates above the grey solid line represent underestimates; those below the grey solid line represent overestimates. The vertical bars represent 95%CIs. LV, left ventricular

In Model 1, the AUC was 0.852 (95% CI 0.771–0.933) in the training set and 0.631 (95% CI 0.421–0.842) in the validation set (Fig. 3). The AUC in Model 2 was similar to that in Model 1 in the training set (0.856, 95% CI 0.781–0.931) but lower in the validation set (0.617, 95% CI 0.406–0.827), while no differences were found in the comparison of two models’ AUC in the training set (*P* = 0.838) and the validation set (*P* = 0.734) (Figure S 2). To be brief, Model 1 demonstrated a stronger capacity to discriminate. Moreover, both models' DCA curves displayed a comparable range of cutoff probabilities, indicating equal clinical efficacy (Figure S3-S4). Model 1 showed a considerably better calibration of the model with more plots surrounding the ideal curves (Fig. 4, Figure S5). Above all, Model 1 was selected because it had a higher AUC-estimated predictive value and a comparable capacity to illustrate the net benefit of clinical decisions. Table 3 shows the outcome of Model 1's prediction after taking the six variables into account. Additionally, by using cross-validation, the accuracy of Model 1 was 0.838 while the Kappa value was 0.416.

### Clinical utility

According to Model 1 (variables included *diagnosis, antiplatelet therapy, thickness of thrombi, thrombus morphology, ventricular aneurysm, and D-dimer levels*), the rate of persistent/recurrent LV thrombus within 3 months increased as thrombus thickness increased (OR 1.11, 95% CI 1.05–1.20, *P* = 0.002). It was interesting to note that patients with protuberant thrombus had a lower rate of thrombus persistence/recurrence compared to those with mural thrombus (OR 0.17, 95% CI 0.03–0.69, *P* = 0.025). Patients with higher baseline D-dimer levels had a lower likelihood of developing persistent or recurrent thrombus at 3 months (OR 0.67, 95% CI 0.43–0.91, *P* = 0.030), and there was a linear relationship between the primary end-point and the thrombus thickness or D-dimer levels (all p for non-linear > 0.05; Figure S6). In addition, the incidence of LV thrombus persistence/recurrence at 3 months differed among patients with different diagnoses or different antiplatelet therapy, and it was uncertain which diagnosis or antiplatelet therapy had more effect on the primary end-point because of their wide CIs.

**Fig. 5:**
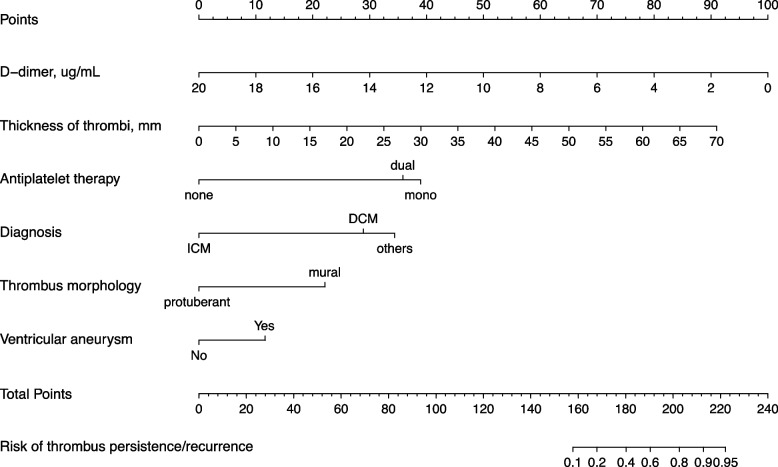
Nomogram for the prediction of LV thrombus persistence/recurrence at 3 months in Model 1. To use the nomogram, an individual patient’s value is located on each variable axis, and a line is drawn upward to determine the number of points received for each variable value. The sum of these numbers is located on the Total Points axis, and a line is drawn downward to the last axis to determine the risk of thrombus persistence/recurrence. LV, left ventricular; ICM, ischemic cardiomyopathy; DCM, dilated cardiomyopathy

Furthermore, we developed a nomogram risk prediction model that comprised independent risk factors (R^2^ 0.38, C index 0.85, 95% CI 0.77–0.93) (Fig. 5). The scores of the items displayed in the nomogram should be added up (Table 4). For example, an echocardiogram revealed a 10 mm thick LV mural thrombus in the ventricular aneurysm in a patient with ICM who had a D-dimer level of 2 ng/mL at admission. During the hospitalization, rivaroxaban plus aspirin treatment was then administered to the patient. According to our prediction model, the overall score of the patient was 176, and the likelihood of LV thrombus persistence/recurrence within 3 months was roughly 40%.

**Table 4:** A risk score for predicting LV thrombus persistence/recurrence at 3 months

**Variable**	**Score**
Diagnosis	
ICM	0
DCM	29
Others	34
Antiplatelet therapy	
None	0
Mono	39
Dual	36
Thickness of thrombi, mm	
5	7
10	13
20	26
30	39
40	52
50	65
60	78
Thrombus morphology	
Mural	22
Protuberant	0
Ventricular aneurysm	
No	0
Yes	12
D-dimer, ug/mL	
2	90
4	80
6	70
8	60
10	50
14	30
18	10
20	0
**Score**	**Prediction probability**
158	10%
168	20%
180	40%
190	60%
203	80%
213	90%
222	95%

*Abbreviations*: *LV* Left ventricular, *ICM* Ischemic cardiomyopathy, *DCM* Dilated cardiomyopathy

### Outcome of thrombus resolution, bleeding, and major cardiovascular events

**Fig. 6:**
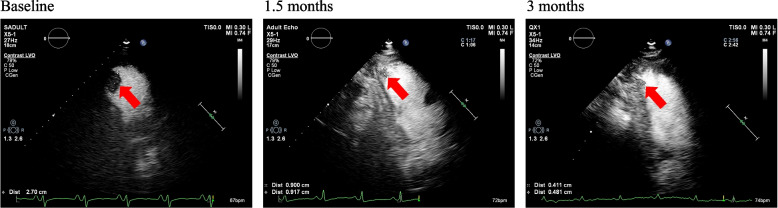
Patient example of CE images. The thrombus is persistent at 1.5 months and 3 months, compared to LV thrombus (red arrows) at baseline. LV, left ventricular; CE, contrast-enhanced echocardiography

At 3 months, 38 patients (22.1%) had persistent or recurrent LV thrombus, compared to a total of 134 patients (77.9%), who had their thrombus resolved. Figure 6 depicted an illustration of a patient with persistent thrombus by CE despite receiving anticoagulant medication for 3 months. Patients in the thrombus resolution group had a higher prevalence of previous heart failure, lower LVEF and higher levels of N-Terminal pro-Brain Natriuretic Peptide (NT-proBNP), a smaller baseline thrombus, a higher proportion of spontaneous echo contrast, and a lower incidence of regional wall motion abnormality and ventricular aneurysm (Table 2).

Three patients experienced bleeding during a 3-month follow-up; one of them reported eye hemorrhage, while the other two experienced nose bleeding. During hospitalization, nine patients suffered major cardiovascular events—six patients reported having a stroke and three patients encountered a pulmonary embolism (Table S1).

## Discussion

It was the first time in a prospective study and a retrospective study to predict the risk of LV thrombus persistence/recurrence among patients with oral anticoagulation for 3 months. The key findings were as follows. Patients who had thicker thrombus, mural thrombus morphology, ventricular aneurysm, or lower D-dimer levels were more likely to have persistent/recurrent LV thrombus. Therefore, irrespective of insufficient evidence, we advocated that anticoagulant therapy could be prolonged and individualized for patients with these high-risk characteristics.

The rate of thrombus persistence/recurrence in the combined study was considerably low thanks to the strict inclusion criteria in the retrospective study and the intensive monitoring in the prospective trial, which demonstrated that patients had relatively fresh thrombi prior to the enrollment and adhered to therapy well over the study period. Another significant aspect was the prospective trial's use of CE at baseline and follow-up visits, which improved the study's power to precisely identify LV thrombus. The incidence of thrombus unresolved was 31.0% in an updated meta-analysis that included 21 studies with 3057 patients over a median follow-up of 12 months [3]. According to a retrospective cohort study, 34.2% of patients with heart failure found persistent LV thrombus with a median duration of 17 months [17]. Based on these studies, it is unclear whether the rate of persistent thrombus will decrease over time given that some persistent thrombi are more likely to be calcified or organized despite anticoagulation in long-term follow-up.

In decades of research, the optimal anticoagulation treatment for LV thrombus has remained controversial. Prior recommendations suggest that anticoagulant therapy with vitamin K antagonists (VKAs) was appropriate for patients with myocardial infarction and asymptomatic LV thrombi for up to 3 months [2, 18]. The 2017 ESC guidelines for myocardial infarction recommended the duration of anticoagulation (either NOACs or VKAs) might be for 6 months guided by repeated imaging [19], in addition, the scientific statement from the American Heart Association (2022) indicated that NOACs were considered to be a reasonable alternative to VKAs in patients with LV thrombus, according to currently available data [3]. The duration of anticoagulation for persistent thrombus, meanwhile, is yet undetermined. According to consensus opinion [3], patients with persistent or recurrent thrombus should continue anticoagulation until resolution on the basis of their high compliance and frequent imaging assessments. A trial of alternative anticoagulation, on the other hand, should be addressed on a case-by-case basis. For those thrombi that are organized or calcified, discontinuing oral anticoagulation is an option because the risk of embolization is probably minimal. Consequently, long-term anticoagulation management options for patients with persistent thrombus should weigh the concerns of indefinite anticoagulation (e.g., increased pill burden and bleeding) against the potential reduction in stroke risk.

We came to the conclusion that patients with persistent thrombus shared six characteristics in addition to presumably poor adherence. It is well acknowledged that patients with different etiologies had different mechanisms in the development of LV thrombus. Based on Virchow's triad of thrombogenesis, three factors include stasis attributable to reduced ventricular function, endocardial injury, and inflammation/hypercoagulability. The interaction between these mechanisms and thrombus outcome, however, is not always easy to clarify [20, 21]. According to our finding, the rate of thrombus persistence/recurrence at 3 months differed among patients with ICM, DCM, and other cardiovascular disorders while it was unreliable to conclude whether DCM or other diseases patients had a higher likelihood of thrombus persistence than those with ICM. Indeed, researchers reported that specific causes of DCM (eg, amyloidosis, eosinophilic myocarditis) could increase the risk of LV thrombus persistent/recurrent [21, 22]. In terms of oral anticoagulation, patients taking NOACs experienced less likelihood of LV thrombus persistence at 3 months than those with VKAs, though the statistical difference was insignificant. A comparable conclusion was reached by several meta-analyses [23–26] and two small randomized clinical trials (apixaban or rivaroxaban versus warfarin) [27, 28]. Interestingly, although the CI was somewhat wide, it allowed us to conclude that patients with mono or dual antiplatelet therapy had a different LV thrombus persistence/recurrence rate at 3 months compared to those without. A prior study [13] reported that patients taking anticoagulation in combination with mono antiplatelet therapy had a higher risk of persistent thrombus than those who did not. In the study of Niku et al. [29], the rate of receiving antiplatelet therapy paired with anticoagulation was higher in patients with persistent left atrial thrombus than those with thrombus resolution (65% vs 38%, *P* = 0.03). Overall, for patients undergoing percutaneous coronary intervention with an indication for antiplatelet therapy and who also have an indication for oral anticoagulation, a general strategy (preferably a NOAC plus clopidogrel) may be considered on the basis of current practice and guideline recommendations [30–32].

In addition, patients with lower D-dimer levels in our analysis had a higher likelihood of LV thrombus persistence/recurrence at 3 months; however, the result would be adjusted by the mean D-dimer level in future work. The reason might be concluded that the thrombus of patients with a low D-dimer was neither fresh nor mobile, resulting in a persistent thrombus. The grade of thrombus mobility was found to be strongly associated with the thrombus' outcome. Limited data suggest that a large or mural thrombus has a less likelihood of thrombus resolution than a small or protuberant thrombus [33]. When evaluating the shape and the morphology of LV thrombus, the findings from Salah et al. [15], which were in agreement with our results, showed that patients with persistent thrombus had bigger baseline thrombus areas. From the consensus of the statement [3], it is unreasonable to give anticoagulation for mural thrombi even though the risk of embolization may be less than for protuberant thrombi. To conclude, a shared decision-making approach is appropriate following a risk/benefit discussion between patients and clinicians.

The major limitations were listed. First, since the sample of this study is small even when combining both the prospective and retrospective cohorts, which limits the power and utility of the model, external validations of our model are expected. Second, due to the wide CIs, it is still unclear whether patients with DCM and antiplatelet therapy are associated with a high risk of LV thrombus persistence/recurrence or not, so additional prospective research is needed to confirm the results. Third, the anticipated medication after 3 months remains unknown in the retrospective study owing to the short-term follow-up, while patients in the prospective study were informed of the appropriate anticoagulant therapy for at least 6 months. Large-scale trials are essential to determine whether indefinite anticoagulation is merited in patients with persistent/recurrent LV thrombus.

## Conclusions

A prediction model comprising six variables was derived from a combination of prospective and retrospective studies. Patients were more likely to develop persistent or recurrent LV thrombus at 3 months if they had thicker thrombus, mural thrombus, ventricular aneurysm, or low baseline D-dimer levels. Considerations on the duration of anticoagulation should be based on the best clinical judgment and shared decision-making, and prospective randomized trials are necessary to validate the model.

## Supplementary Information

**Additional file 1:** **Fig. S1.** LASSO coefficient profiles of the 44 variables. A coefficient profile plot was produced against the log (Lambda) sequence. **Fig. S2.** ROC curves of Model 1 and Model 2 for predicting the risk of LV thrombus persistence/recurrence at 3 months. (A) Training set. (B) Validation set. The black curve represents the model discrimination ability of Model 1 either in the training set or the validation set. The red curve represents the model discrimination ability of Model 2 either in the training set or the validation set. The grey curve represents that a model has no model discrimination ability. No differences were found in the comparison of the two models’ AUC in the training set (*P* = 0.838) and the validation set (*P* = 0.734). LV, left ventricular; ROC, receiver operating characteristic; AUC, area under the ROC. **Fig. S3.** DCA of the nomogram for predicting the risk of LV thrombus persistence/recurrence at 3 months in Model 1. (A) Training set. (B) Validation set. X-axis: cut-off probability; Y-axis: net benefit, which is calculated across a range of threshold probabilities. The blue line represents Model 1. The grey line represents the assumption that all patients have thrombus persistence/recurrence. The black line represents the assumption that no patients have thrombus persistence/recurrence. LV, left ventricular; DCA, decision curve analysis. **Fig. S4.** DCA of the nomogram for predicting the risk of LV thrombus persistence/recurrence at 3 months in Model 1 and Model 2. (A) Training set. (B) Validation set. X-axis: cut-off probability; Y-axis: net benefit, which is calculated across a range of threshold probabilities. The blue line represents Model 1. The red line represents Model 2. The grey line represents the assumption that all patients have thrombus persistence/recurrence. The black line represents the assumption that no patients have thrombus persistence/recurrence. LV, left ventricular; DCA, decision curve analysis. **Fig. S5.** Calibration plots for predicting the risk of LV thrombus persistence/recurrence at 3 months in Model 2. (A) Training set. (B) Validation set. X-axis: predicted thrombus persistence/recurrence risk; Y-axis: actual thrombus persistence/recurrence rate. Estimates above the grey solid line represent underestimates; those below the grey solid line represent overestimates. The vertical bars represent 95%CIs. LV, left ventricular; CI, confidence interval. **Fig. S6.** Restricted cubic spline curve. We observed a linear relationship between LV thrombus persistence/recurrence at 3 months and continuous variables including (A) D-dimer levels and (B) thickness of thrombi (all p for nonlinear 0.05). The p values for overall association were less than 0.05 for thrombus persistence/recurrence. Both models were adjusted for cofounders in Model 1 including diagnosis, antiplatelet therapy, thrombus morphology, and ventricular aneurysm. ORs are indicated by solid lines and 95%CIs by shaded areas. LV, left ventricular; OR, odds ratio; CI, confidence interval. **Table S1.** Secondary outcomes at 3-month follow-up [N = 172].

## Data Availability

The data will be shared on reasonable request with the corresponding author.
