# Supplementary material for: A prediction model for left ventricular thrombus persistence/recurrence: based on a prospective study and a retrospective study
Source: Thromb J. 2023 May 1;21:50. doi: 10.1186/s12959-023-00488-1 (PMC10150477; doi:10.1186/s12959-023-00488-1)
Supplement: Supplementary file 1 — Additional file 1: Fig. S1. LASSO coefficient profiles of the 44 variables. A coefficient profile plot was produced against the log (Lambda) sequence. Fig. S2. ROC curves of Model 1 and Model 2 for predicting the risk of LV thrombus persistence/recurrence at 3 months. (A) Training set. (B) Validation set. The black curve represents the model discrimination ability of Model 1 either in the training set or the validation set. The red curve represents the model discrimination ability of Model 2 either in the training set or the validation set. The grey curve represents that a model has no model discrimination ability. No differences were found in the comparison of the two models’ AUC in the training set (P = 0.838) and the validation set (P = 0.734). LV, left ventricular; ROC, receiver operating characteristic; AUC, area under the ROC. Fig. S3. DCA of the nomogram for predicting the risk of LV thrombus persistence/recurrence at 3 months in Model 1. (A) Training set. (B) Validation set. X-axis: cut-off probability; Y-axis: net benefit, which is calculated across a range of threshold probabilities. The blue line represents Model 1. The grey line represents the assumption that all patients have thrombus persistence/recurrence. The black line represents the assumption that no patients have thrombus persistence/recurrence. LV, left ventricular; DCA, decision curve analysis. Fig. S4. DCA of the nomogram for predicting the risk of LV thrombus persistence/recurrence at 3 months in Model 1 and Model 2. (A) Training set. (B) Validation set. X-axis: cut-off probability; Y-axis: net benefit, which is calculated across a range of threshold probabilities. The blue line represents Model 1. The red line represents Model 2. The grey line represents the assumption that all patients have thrombus persistence/recurrence. The black line represents the assumption that no patients have thrombus persistence/recurrence. LV, left ventricular; DCA, decision curve analysis. Fig. S5. Calibration p [file 12959_2023_488_MOESM1_ESM.docx]

**A prediction model for left ventricular thrombus persistence/recurrence: based on a prospective study and a retrospective study**

[Figure and Table Legends 2](#_Toc128908561)

[Supplementary Figure 3](#_Toc128908562)

[Supplementary Table 9](#_Toc128908563)

Figure and Table Legends

**Fig.S1 LASSO coefficient profiles of the 44 variables.** A coefficient profile plot was produced against the log (Lambda) sequence.

**Fig.S2 ROC curves of Model 1 and Model 2 for predicting the risk of LV thrombus persistence/recurrence at 3 months.** (A) Training set. (B) Validation set. The black curve represents the model discrimination ability of Model 1 either in the training set or the validation set. The red curve represents the model discrimination ability of Model 2 either in the training set or the validation set. The grey curve represents that a model has no model discrimination ability. No differences were found in the comparison of the two models’ AUC in the training set (P = 0.838) and the validation set (P = 0.734). LV, left ventricular; ROC, receiver operating characteristic; AUC, area under the ROC.

**Fig.S3** **DCA of the nomogram for predicting the risk of LV thrombus persistence/recurrence at 3 months in Model 1.** (A) Training set. (B) Validation set. X-axis: cut-off probability; Y-axis: net benefit, which is calculated across a range of threshold probabilities. The blue line represents Model 1. The grey line represents the assumption that all patients have thrombus persistence/recurrence. The black line represents the assumption that no patients have thrombus persistence/recurrence. LV, left ventricular; DCA, decision curve analysis.

**Fig.S4** **DCA of the nomogram for predicting the risk of LV thrombus persistence/recurrence at 3 months in Model 1 and Model 2.** (A) Training set. (B) Validation set. X-axis: cut-off probability; Y-axis: net benefit, which is calculated across a range of threshold probabilities. The blue line represents Model 1. The red line represents Model 2. The grey line represents the assumption that all patients have thrombus persistence/recurrence. The black line represents the assumption that no patients have thrombus persistence/recurrence. LV, left ventricular; DCA, decision curve analysis.

**Fig.S5 Calibration plots for predicting the risk of LV thrombus persistence/recurrence at 3 months in Model 2.** (A) Training set. (B) Validation set. X-axis: predicted thrombus persistence/recurrence risk; Y-axis: actual thrombus persistence/recurrence rate. Estimates above the grey solid line represent underestimates; those below the grey solid line represent overestimates. The vertical bars represent 95%CIs. LV, left ventricular; CI, confidence interval.

**Fig.S6 Restricted cubic spline curve.** We observed a linear relationship between LV thrombus persistence/recurrence at 3 months and continuous variables including (A) D-dimer levels and (B) thickness of thrombi (all p for nonlinear > 0.05). The p values for overall association were less than 0.05 for thrombus persistence/recurrence. Both models were adjusted for cofounders in Model 1 including diagnosis, antiplatelet therapy, thrombus morphology, and ventricular aneurysm. ORs are indicated by solid lines and 95%CIs by shaded areas. LV, left ventricular; OR, odds ratio; CI, confidence interval.

**Table S1.** Secondary outcomes at 3-month follow-up [N = 172]

**Abbreviations:** N, number of patients, CI, confidence interval.

Supplementary Figure

**Fig.S1 LASSO coefficient profiles of the 44 variables.** A coefficient profile plot was produced against the log (Lambda) sequence.

(B)

(A)

**Fig.S2 ROC curves of Model 1 and Model 2 for predicting the risk of LV thrombus persistence/recurrence at 3 months.** (A) Training set. (B) Validation set. The black curve represents the model discrimination ability of Model 1 either in the training set or the validation set. The red curve represents the model discrimination ability of Model 2 either in the training set or the validation set. The grey curve represents that a model has no model discrimination ability (AUC = 0.500). No differences were found in the comparison of the two models’ AUC in the training set (P = 0.838) and the validation set (P = 0.734). LV, left ventricular; ROC, receiver operating characteristic; AUC, area under the ROC.

(A)

(B)

**Fig.S3** **DCA of the nomogram for predicting the risk of LV thrombus persistence/recurrence at 3 months in Model 1.** (A) Training set. (B) Validation set. X-axis: cut-off probability; Y-axis: net benefit, which is calculated across a range of threshold probabilities. The blue line represents Model 1. The grey line represents the assumption that all patients have thrombus persistence/recurrence. The black line represents the assumption that no patients have thrombus persistence/recurrence. LV, left ventricular; DCA, decision curve analysis.

(A)

(B)

**Fig.S4** **DCA of the nomogram for predicting the risk of LV thrombus persistence/recurrence at 3 months in Model 1 and Model 2.** (A) Training set. (B) Validation set. X-axis: cut-off probability; Y-axis: net benefit, which is calculated across a range of threshold probabilities. The blue line represents Model 1. The red line represents Model 2. The grey line represents the assumption that all patients have thrombus persistence/recurrence. The black line represents the assumption that no patients have thrombus persistence/recurrence. LV, left ventricular; DCA, decision curve analysis.

(A)

(B)

**Fig.S5 Calibration plots for predicting the risk of LV thrombus persistence/recurrence at 3 months in Model 2.** (A) Training set. (B) Validation set. X-axis: predicted thrombus persistence/recurrence risk; Y-axis: actual thrombus persistence/recurrence rate. Estimates above the grey solid line represent underestimates; those below the grey solid line represent overestimates. The vertical bars represent 95%CIs. LV, left ventricular; CI, confidence interval.

**Fig.S6 Restricted cubic spline curve.** We observed a linear relationship between LV thrombus persistence/recurrence at 3 months and continuous variables including (A) D-dimer levels and (B) thickness of thrombi (all p for non-linear > 0.05). The p values for overall association were less than 0.05 for thrombus persistence/recurrence. Both models were adjusted for cofounders in Model 1 including diagnosis, antiplatelet therapy, thrombus morphology, and ventricular aneurysm. ORs are indicated by solid lines and 95%CIs by shaded areas. LV, left ventricular; OR, odds ratio; CI, confidence interval.

Supplementary Table

**Table S1.** Secondary outcomes at 3-month follow-up [N = 172]

|  | **N** | **%** | **95% CI** |
| --- | --- | --- | --- |
| **Thrombus resolution** | 134 | 77.9 | 71.0-83.9 |
| **Safety outcome** | 3 | 1.7 | 0.4-5.0 |
| Major bleeding | 1 | 0.6 | 0.01-3.2 |
| Clinically relevant non-major bleeding | 2 | 1.2 | 0.1-4.1 |
| **Stroke/embolism** | 9 | 5.2 | 2.4-9.7 |

**Abbreviations:** N, number of patients, CI, confidence interval.
